# P-1710. Epidemiology and Outcomes of Non-Albicans Candidemia in a Tertiary Hospital in the Dominican Republic

**DOI:** 10.1093/ofid/ofaf695.1882

**Published:** 2026-01-11

**Authors:** Alexis A Cuevas, Ramon Romano, Elianet Castillo, Claudia Blanco, Alfredo J Mena Lora

**Affiliations:** CEDIMAT, Santo Domingo, Distrito Nacional, Dominican Republic; CEDIMAT, Santo Domingo, Distrito Nacional, Dominican Republic; CEDIMAT/CEMDOE, Santo Domingo, Distrito Nacional, Dominican Republic; SDI, Santo Domingo, Distrito Nacional, Dominican Republic; University of Illinois Chicago, Chicago, Illinois

## Abstract

**Background:**

Candidemia is a life-threatening fungal infection with a mortality rate of ∼25% in the U.S., rising to 60% worldwide. In Latin America, mortality reaches 50%. The incidence of non-albicans Candida (NAC) is rising. Although clinical presentation is often similar, NAC species can be intrinsically or secondarily resistant to antifungals. Despite high mortality, data on candidemia in the Dominican Republic are lacking. This study aims to describe the epidemiology, characteristics, and outcomes of candidemia at a tertiary center, highlighting the growing threat of NAC.

**Figure:**
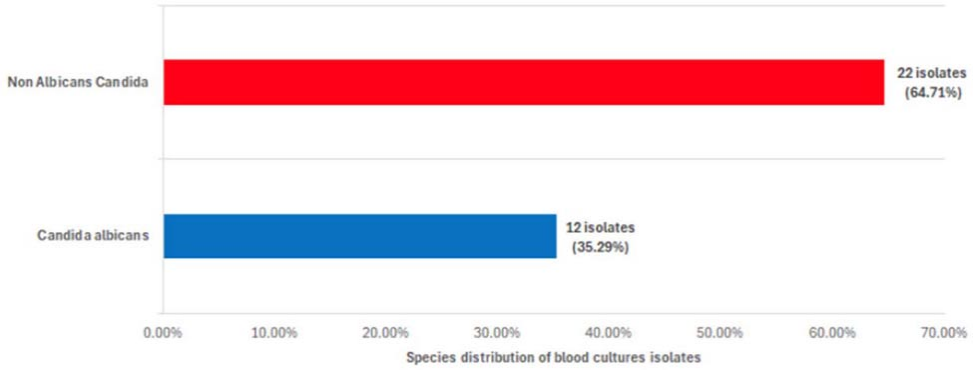
Species distribution of candidemia

**Table 1:**
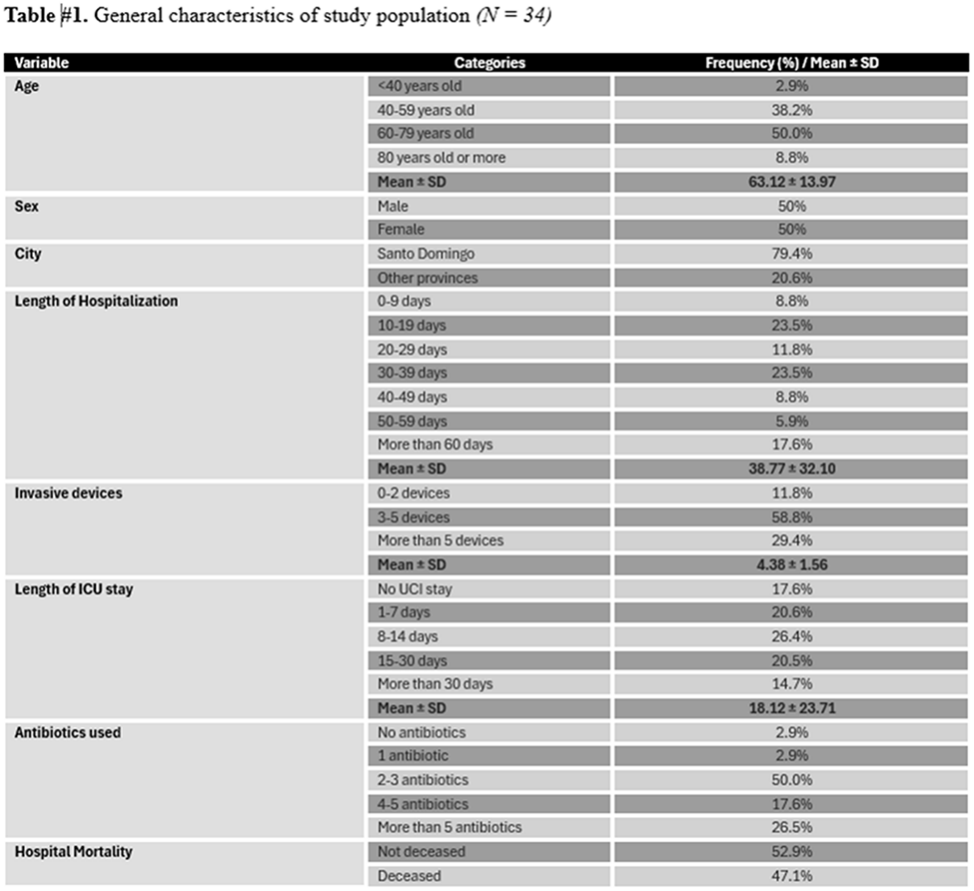
Clinical characteristics

**Methods:**

A retrospective, observational study was conducted at CEDIMAT. Records of hospitalized patients with candidemia (Jan 2021–Dec 2024) were reviewed. Demographic, clinical, microbiological data and outcomes were collected. Descriptive and inferential analyses, including logistic regression and ROC curves, were used.

**Results:**

Thirty-four patients were analyzed. NAC species accounted for 64.7%, including *C. glabrata*, *C. parapsilosis*, *C. guilliermondii*, and *C. ciferrii*. Overall mortality was 47.1%. Deceased patients were older (65.1 vs. 59.8 yrs, p=0.030) and had longer ICU stays (21.3 vs. 14.2 days, p=0.012). Mechanical ventilation was strongly associated with mortality (83.3%, p< 0.001). Persistent candidemia was associated with a higher Charlson comorbidity index (p=0.034). Use of multiple invasive devices showed a trend toward persistent candidemia (OR 2.48, CI 0.92–4.98, p=0.051). ROC curves showed strong model performance (AUC 0.903 for mortality; 0.969 for persistence).

**Conclusion:**

Candidemia in this cohort had high mortality, driven by NAC species and resistance. Age, ICU stay, ventilation, and persistent fungemia were key factors. Early interventions, strict infection control, and preventive strategies are warranted. Further research is needed to guide regional management and improve outcomes.

**Disclosures:**

All Authors: No reported disclosures

